# Self-supervised MRI denoising: leveraging Stein’s unbiased risk estimator and spatially resolved noise maps

**DOI:** 10.1038/s41598-023-49023-2

**Published:** 2023-12-19

**Authors:** Laura Pfaff, Julian Hossbach, Elisabeth Preuhs, Fabian Wagner, Silvia Arroyo Camejo, Stephan Kannengiesser, Dominik Nickel, Tobias Wuerfl, Andreas Maier

**Affiliations:** 1 Pattern Recognition Lab, Friedrich-Alexander-Universität Erlangen-Nürnberg, 91058 Erlangen, Germany; 2 Magnetic Resonance, Siemens Healthcare GmbH, 91052 Erlangen, Germany

**Keywords:** Magnetic resonance imaging, Biomedical engineering, Computer science

## Abstract

Thermal noise caused by the imaged object is an intrinsic limitation in magnetic resonance imaging (MRI), resulting in an impaired clinical value of the acquisitions. Recently, deep learning (DL)-based denoising methods achieved promising results by extracting complex feature representations from large data sets. Most approaches are trained in a supervised manner by directly mapping noisy to noise-free ground-truth data and, therefore, require extensive paired data sets, which can be expensive or infeasible to obtain for medical imaging applications. In this work, a DL-based denoising approach is investigated which operates on complex-valued reconstructed magnetic resonance (MR) images without noise-free target data. An extension of Stein’s unbiased risk estimator (SURE) and spatially resolved noise maps quantifying the noise level with pixel accuracy were employed during the training process. Competitive denoising performance was achieved compared to supervised training with mean squared error (MSE) despite optimizing the model without noise-free target images. The proposed DL-based method can be applied for MR image enhancement without requiring noise-free target data for training. Integrating the noise maps as an additional input channel further enables the regulation of the desired level of denoising to adjust to the preference of the radiologist.

## Introduction

Sufficient signal-to-noise ratio (SNR) is a key requirement for achieving diagnostic image quality in magnetic resonance imaging (MRI). Especially for images acquired with low-field scanners (0.25–1 T), low SNR is a common problem which can result in reduced clinical value^1^. In complex-valued reconstructed magnetic resonance (MR) images, the noise can be modeled as a spatially variant Gaussian distribution^2^ and may be determined with the help of a noise adjustment scan^3^. The spatial noise dependence arises due to the anisotropic noise amplification during the image reconstruction process, especially in the context of parallel imaging. In MRI, it is common practice to use magnitude images for diagnostic purposes, where accurately modeling the noise distribution is more complex. Depending on the image acquisition and reconstruction technique, the noise in magnitude images can be modeled, e.g., by a Rician, Rayleigh or non-central Chi distribution^4^.

The appearance of noise does not only affect the visual impression of MR images. Many processing techniques, such as image segmentation or registration, can profit from higher SNR in order to achieve optimal results for the respective task at hand^2^. There are two main directions of noise reduction techniques: acquisition-based signal increase and post-acquisition noise reduction during or after reconstruction. During image acquisition, an improvement of the SNR can either be achieved by averaging over repeated measurements and, thus, extending the acquisition time or by enlarging the voxel size to increase the signal strength^5^. However, in practice the image acquisition time is restricted due to various factors, including scanner throughput and patient comfort. The use of larger voxels further limits spatial resolution. Therefore, a practical upper limit of SNR exists for most MRI applications. Denoising the acquired image using specified algorithms during reconstruction, e.g. via compressed sensing^6^, filtering the image after reconstruction, or a combination of both, are low-cost and efficient alternatives^7^. Common image denoising strategies employed in MRI involve techniques like wavelet-based denoising and non-local means^8^. In non-structural MRI applications such as diffusion-weighted imaging (DWI), where multiple image repetitions capture distinct image contrasts, denoising techniques based on principal component analysis (PCA) further demonstrate practical utility^9–12^.

Over the last few years, deep learning (DL)-based approaches for image denoising achieved state-of-the-art performance by deriving complex features from large amounts of data^13^. Supervised DL approaches require pairs of noisy and noise-free images for training. Here, the primary research effort has been focused on the design of novel network architectures. For instance, Wagner et al.^14^ implemented a robust computed tomography (CT) denoising framework based on trainable bilateral filter layers. Zhang et al.^15^ proposed integrating a spatially variant noise map as additional input channel to the network to further leverage the denoising performance on different noise levels during inference.

However, obtaining strictly noise-free images is not feasible in the context of MRI. For such cases, unsupervised learning methods can be applied, which circumvent the need for paired ground-truth images during model training. Noise2Noise^16^ calculates a loss metric using two independent noisy image realizations, while Noise2Void^17^ employs a blind-spot technique aiming to predict masked image patches. Wagner et al. introduced Noise2Contrast, a self-supervised technique that leverages information from various measured MR image contrasts to train a denoising model^18^. Other methods consider the integration of additional prior information to obtain results that are closer to those of supervised training. Several previous works proposed to employ Stein’s unbiased risk estimator (SURE) as a loss function to approximate the mean squared error (MSE) between output image and unknown ground truth by incorporating the variance of the Gaussian noise distribution for various computer vision applications^13,19–21^.

Recently, diffusion models, a class of generative models that transform random noise into representative data samples, have become increasingly popular in the field of MRI denoising. Xiang et al.^22^ introduced a thee-stage self-supervised denoising approach for diffusion-weighted MRI using diffusion denoising generative models, while Chung et al.^23^ proposed an MRI denoising technique utilizing score-based reverse diffusion sampling.

In MRI, common practice also employs regularization techniques to improve the SNR already during reconstruction^24^. Zhussip et al.^20^ trained a denoising network for compressive MR image recovery from undersampled measurements. The authors first pre-trained their network in a supervised setting using BM3D^25^ and subsequently refined it without ground-truth data using SURE. Similarly, Aggarwal et al.^21^ investigated the reconstruction of MR images from noisy undersampled measurements using SURE.

To our knowledge, in the context of MRI the presented methods based on SURE have so far only been applied considering spatially invariant Gaussian noise. Combining the ability to obtain quantitative noise maps with previous work on the removal of spatially invariant Gaussian noise, in this work,

1. the SURE approach is extended to deal with spatially variant noise in MRI, focusing on conventional 2D TSE acquisitions.
2. we conduct extensive experiments on simulated and real patient MRI scans to prove the effectiveness of our proposed method.
3. we demonstrate how to control the tradeoff between denoising and image sharpness by using a model conditioned on the noise map.

## Theory

### Noise estimation in MRI

The main source of noise in MRI is thermal noise, which originates from the subject being scanned, as well as from the electronic components in the receiver chain^26^.

**Figure 1:**
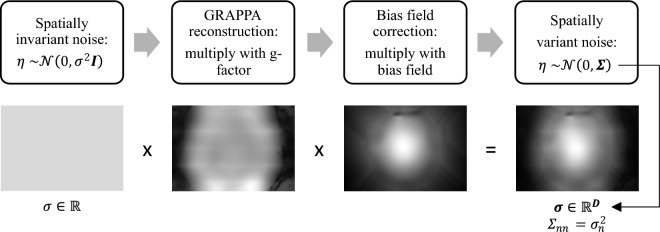
The process for calculating the noise map. The spatially invariant thermal noise distribution is propagated through the image reconstruction pipeline, resulting in a spatially variant Gaussian distribution with a pixel-wise standard deviation described by noise map $\varvec{\sigma }$.

The noise can be modelled as an additive, uncorrelated contribution to the pure MR signal that follows a Gaussian distribution in the real and imaginary part of complex-valued reconstructed images^26^. The use of parallel imaging techniques further introduces a non-uniform noise enhancement, resulting in a spatially variant Gaussian noise distribution^2^. A noise map specifying the standard deviation of the noise for every pixel of the final image can be obtained by propagating the original spatially invariant noise distribution through all steps of the image reconstruction pipeline, similar to Kellman et al.^3^. We focus on the noise propagation for conventional parallel imaging as performed by the scanner integrated reconstruction. Based on a noise adjustment scan the thermal noise level and decorrelation matrices are determined. For the actual image reconstruction this information can be retrieved and starting from decorrelated *k*-space data with univariate Gaussian noise, the noise map is determined by propagation through the sequential reconstruction steps, resulting in complex-valued images with a Gaussian noise distribution and known standard deviation for each pixel. The overall process is illustrated in Fig. 1.

First, the standard deviation of each *k*-space sample originating from the thermal noise distribution is determined through a rapid noise calibration scan routinely performed without radiofrequency excitation as part of the scanner adjustments^3^. Second, the change in the noise distribution due to the reconstruction process is considered, including *k*-space filtering and scaling by Fourier transforms. The spatially variant noise enhancement is influenced by the coil geometry factor, referred to as g-factor. For generalized autocalibrating partially parallel acquisitions (GRAPPA)^27^, this g-factor can be derived directly from the GRAPPA reconstruction weights and channel combination coefficients, as proposed by Breuer et al.^28^. To define the noise distribution of the resulting reconstructed images, the original standard deviation is multiplied with the noise enhancement from the GRAPPA reconstruction.

A subsequent step in the image reconstruction pipeline is the surface coil intensity correction, as described in^29^. Surface coils placed around the patient demonstrate higher sensitivity near the surface of the body, whereas signals coming from more distant regions inside the body appear attenuated. The resulting intensity variations are corrected by multiplying the images with the corresponding bias field, which can be estimated with a separate scan prior to the measurement. Therefore, the spatially resolved noise map is a multiplicative combination of the initial standard deviation, the g-factor, and the bias field. Note that a noise decorrelation step is incorporated into the processing pipeline and performed prior to reconstruction, such that the noise correlation matrix $\Sigma$ becomes diagonal^3,30,31^.

### SURE

#### Conventional SURE approach for spatially invariant Gaussian noise

SURE was first proposed by Charles Stein in 1981^32^ and provides a statistical method to estimate the MSE between the unknown mean ${\textbf {x}}\in {{\mathbb {R}}}^{D}$ of a multivariate Gaussian distributed signal ${\textbf {y}}\in {{\mathbb {R}}}^{D}$ and its estimate ${\hat{{\textbf {x}}}}=f({\textbf {y}})$. This can be adapted to tackle image denoising problems as shown by Metzler et al.^19^. Here, the goal is to reconstruct an unknown noise-free image ${\textbf {x}}$ corrupted by Gaussian noise $\varvec{\eta }\sim \ {\mathscr {N}}(0,\sigma ^2 {\textbf {I}})$, where ${\textbf {I}}$ denotes the identity matrix, from a noisy image ${\textbf {y}}= {\textbf {x}}+ \varvec{\eta }$. Since the noise is additive and has zero mean, the unknown noise-free image ${\textbf {x}}$ can be considered as the mean vector of the noisy image ${\textbf {y}}$, such that ${\textbf {y}}\sim \ {\mathscr {N}}({\textbf {x}},\sigma ^2 {\textbf {I}})$. For the denoising task we can now train a neural network $f({\textbf {y}})$ that receives noisy measurements ${\textbf {y}}$ as input and predicts an estimate of ${\textbf {x}}$ as output using the expectation of the MSE expressed as:

1 $$\begin{aligned} \text {E}_{{\textbf {x}}}\Big \{\ \hspace{-0.3em} \frac{1}{D} \Vert f({\textbf {y}})-{\textbf {x}}\Vert ^2 \Big \}\ =&\, \, \text {E}_{{\textbf {x}}}\Big \{\ \hspace{-0.3em} \frac{1}{D} \Vert f({\textbf {y}})-{\textbf {y}}\Vert ^2 - \sigma ^2 + \frac{2}{D}\sigma ^2 \text {div}_{{\textbf {y}}}(f({\textbf {y}}))\Big \}\ , \end{aligned}$$

with

2 $$\begin{aligned} \text {div}_{{\textbf {y}}}(f({\textbf {y}}))= \sum \limits _i^D \frac{\partial f_i({\textbf {y}})}{\partial y_i}\hspace{5.0pt}. \end{aligned}$$

In practice, the expected value in Eq. (1) can be approximated by averaging the loss over several images within a batch. The first term, $\frac{1}{D}\Vert f({\textbf {y}})-{\textbf {y}}\Vert ^2$, is denoted as *fidelity term* and minimizes the difference between the observation (input) and estimation (output) of the denoiser. If the network was only trained with this term, it would simply learn the identity mapping. In contrast, the *divergence term* $\text {div}_{{\textbf {y}}}(f({\textbf {y}}))$ penalizes the model for varying its predictions in the case that there are minor changes in the observations and thus acts as a counterpart to the *fidelity term*^21^.

Especially for advanced denoising operators it is often hard or even impossible to calculate the *divergence term* analytically. In the case of a neural network, the computation of the divergence, i.e., the trace of the Jacobian matrix of the network output with respect to the input, would be time-consuming and inefficient. To solve this problem, Ramani et al. proposed MC-SURE, a Monte Carlo (MC) method that provides an estimate of the *divergence term*^33^. Let ${\textbf {b}}$ be a zero-mean i.i.d. random vector with unit variance and $\epsilon$ is a fixed small value that can, e.g., be defined as $\epsilon =\max ({\textbf {y}})\times 10^{-3}$. Then

3 $$\begin{aligned} \text {div}_{{\textbf {y}}}(f({\textbf {y}}))= \lim \limits _{\epsilon \rightarrow 0} \text {E}_{{\textbf {b}}}\bigg \{\ \hspace{-0.3em} {\textbf {b}}^T \bigg (\ \frac{f({\textbf {y}}+\epsilon {\textbf {b}})-f({\textbf {y}})}{\epsilon } \bigg )\ \hspace{-0.4em} \bigg \}\ . \end{aligned}$$

Considering the law of large numbers, it is reasonable to assume that taking only a single realization of ${\textbf {b}}$ will result in a sufficiently accurate estimation of the variance^33^. Taking this approximation into consideration, the following formulation applies:

4 $$\begin{aligned} \text {div}_{{\textbf {y}}}(f({\textbf {y}}))\approx {\textbf {b}}^T \bigg (\ \hspace{-0.3em} \frac{f({\textbf {y}}+\epsilon {\textbf {b}})-f({\textbf {y}})}{\epsilon } \bigg )\ . \end{aligned}$$

By combining SURE with the MC divergence estimation, it is possible to minimize a fairly accurate estimation of the MSE loss between a denoised image and its unknown noise-free ground truth. The *fidelity term* ensures that the network output does not deviate too much from the input, while the *divergence term* prevents the network from learning the identity mapping. The original SURE formula only considered the estimation of the mean of a Gaussian distributed vector. Over the last years, many extensions have been proposed for other distributions^34,35^.

#### Extended SURE approach for spatially variant Gaussian noise

In order to properly address the spatially variant noise enhancement in reconstructed MR images, we extended the MC-SURE approach accordingly. Instead of the scalar variance $\sigma ^2$, we incorporate a noise map $\varvec{\sigma } \in {{\mathbb {R}}}^{D}$, indicating the standard deviation of the noise for every pixel^36^.

The noisy measurement vector ${\textbf {y}}$ follows a multivariate Gaussian distribution with clean image ${\textbf {x}}$ as mean and covariance matrix $\varvec{\Sigma }$ modelling the additive noise $\varvec{\eta }\sim \ {\mathscr {N}}(0,\varvec{\Sigma })$. We consider the noise to be independently *but not identically* distributed for each pixel, therefore $\varvec{\Sigma }$ can be interpreted as a diagonal matrix with entries $\Sigma _{dd} = \sigma _d^2$, where $\sigma _i$ are the components of the noise map $\varvec{\sigma } \in {{\mathbb {R}}}^{D}$. This means that every pixel is corrupted by a distinct Gaussian noise distribution $\eta _d \sim \ {\mathscr {N}}(0,\sigma _d)$.

Suppose $f({\textbf {y}})$ is an estimator of the unknown ground truth ${\textbf {x}}$ from ${\textbf {y}}$. According to our derivation included in “Appendix”, the expectation of the MSE for spatially variant noise can be expressed using SURE:

5 $$\begin{aligned} \text {E}_{{\textbf {x}}}\Big \{\ \hspace{-0.3em} \frac{1}{D} \Vert f({\textbf {y}})-{\textbf {x}}\Vert ^2 \Big \}\ =&\, \, \text {E}_{{\textbf {x}}}\Big \{\ \hspace{-0.3em} \frac{1}{D} \Bigl ( \Vert f({\textbf {y}})-{\textbf {y}}\Vert ^2 - \sum \limits _d^D\sigma _d^2 + 2 \hspace{0.2em} \text {div}_{{\textbf {y}}} ( \varvec{\sigma ^2} \odot f({\textbf {y}})) \Bigr ) \Big \}\ , \end{aligned}$$

where $\odot$ denotes element-wise multiplication. In practice, we can approximate the expected value by averaging the loss over several images within a processed batch to ensure numerical stability.

Similarly, the MC estimator was adjusted to work with the spatially variant Gaussian SURE formulation following the steps presented by Ramani et al.^33^. Define ${\textbf {b}}\in {{\mathbb {R}}}^{D}$ as a zero-mean i.i.d. random vector with unit variance. Then:

6 $$\begin{aligned} \text {div}_{{\textbf {y}}} ( \varvec{\sigma ^2} \odot f({\textbf {y}})) \approx {\textbf {b}}^T \bigg (\ \hspace{-0.3em} \varvec{\sigma ^2} \odot \Big (\ \frac{f({\textbf {y}}+\epsilon {\textbf {b}})-f({\textbf {y}})}{\epsilon } \Big )\ \hspace{-0.4em} \bigg )\ . \end{aligned}$$

**Figure 2:**
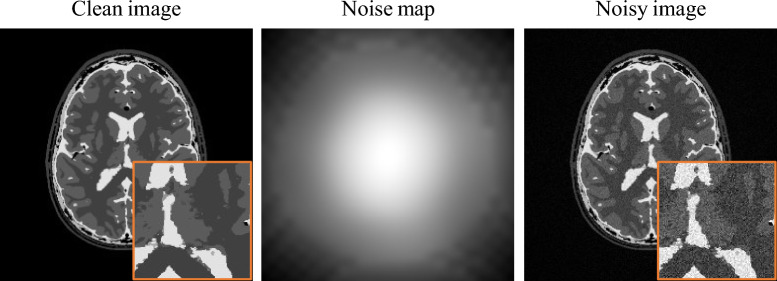
Noisy image ${\varvec{y}}$ was generated from clean image ${\varvec{x}}$ using noise map $\varvec{\sigma }$ indicating the noise level for every pixel.

## Data

### Simulated MRI data

A proper comparison between MSE and SURE requires pairs of clean and noisy images, as well as the corresponding noise maps. This can be realized by taking noise-free images and adding simulated Gaussian noise. However, capturing entirely noise-free MR images is not feasible in practice, as a certain level of noise remains even at high field strengths and long acquisition times. To have full control over the noise that is actually present in the images, we used the simulated *BrainWeb20* MR data base^37^, consisting of 20 anatomical models of normal brains, for our experiments. In total, a set of 2560 noise-free T2-weighted slice images was generated using the *brainweb* Python package.

To achieve a realistic noise simulation, we incorporated 20 dedicated sets of noise maps, considering the spatially variant noise enhancement due to the parallel image reconstruction technique^28^ and the intensity inhomogeneity correction^29^ as described in “Noise estimation in MRI”. The respective noise map $\varvec{\sigma }$ indicating the noise level for every pixel was then multiplied with a Gaussian distributed random vector $\varvec{\xi } \sim \ {\mathscr {N}}(0,1)$ and added to clean image ${\varvec{x}}$ to create noisy image ${\varvec{y}}$. An exemplary simulation is illustrated in Fig. 2.

The simulated *BrainWeb20* data set was split into fixed training, validation, and test sets with 12 anatomical brain models for training and four for validation and testing, respectively.

### Real MRI data

To verify the applicability of our proposed approach on real data, we employed a second data set consisting of 23 T2-weighted head scans (575 slices) measured at a field strength of 0.55 T (MAGNETOM Free.Max, Siemens Healthcare, Erlangen, Germany). The data set was collected in complex-valued reconstructed form with the corresponding noise maps indicating the standard deviation of the noise distribution for every pixel, which were calculated as described in “Noise estimation in MRI”. The data set was split into 19 scans for training and two scans each for validation and testing. We further employed a proton density (PD)-weighted knee scan acquired at 1.5 T (MAGNETOM Sola, Siemens Healthcare, Erlangen, Germany) to assess the robustness of our model with respect to different field strengths, contrasts, and body regions.

The MR measurements were conducted in strict accordance with all relevant ethical regulations, including the principles embodied in the declaration of Helsinki, as well as EU and German law. The responsible licensing authorities, i.e., the Berufsgenossenschaft Energie Textil Elektro Medienerzeugnisse (BG ETEM, IK 120590446) Cologne, Germany, as well as the Gewerbeaufsichtsamt (Regierung von Mittelfranken) approved the volunteer scanning for research purposes under the registration number QR24-03_MR. All participants received an informed consent discussion and gave their written informed consent for their data being further used and processed.

## Experiments and results

**Figure 3:**
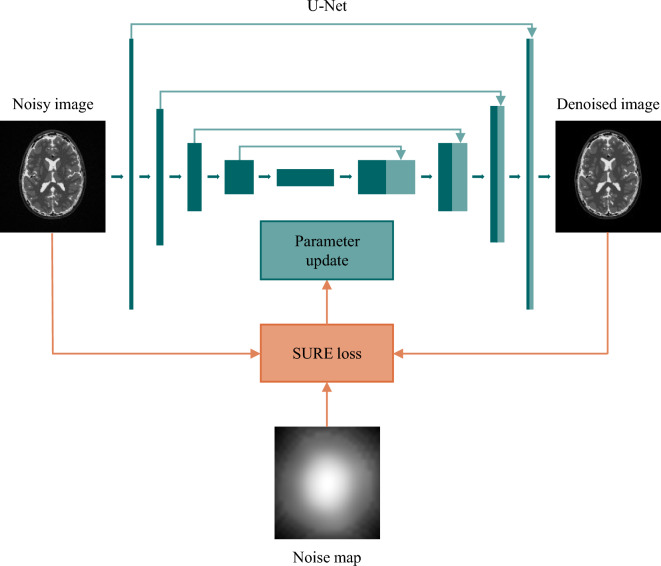
Overview of the training process. The model takes the noisy image as input and produces a denoised image as output. The SURE loss is calculated by considering the noisy input image, the denoised image, and the pixel-wise noise standard deviation map. This loss is subsequently utilized to update the model parameters.

For all our experiments, we trained a U-Net^38^ architecture without BatchNorm layers in PyTorch using the Adam optimizer with default parameters, learning rate $5\times 10^{-5}$, and minibatch size of 8. The training was terminated after 500 epochs. The training process is illustrated in Fig. 3.

### Comparing SURE and MSE

This experiment aimed to determine if SURE can indeed be employed to accurately approximate training with MSE loss without ground-truth data. For evaluation purposes, the metrics MSE, peak signal-to-noise ratio (PSNR) and structural similarity index measure (SSIM) were calculated in 2D and and subsequently averaged across all test slices.

**Figure 4:**
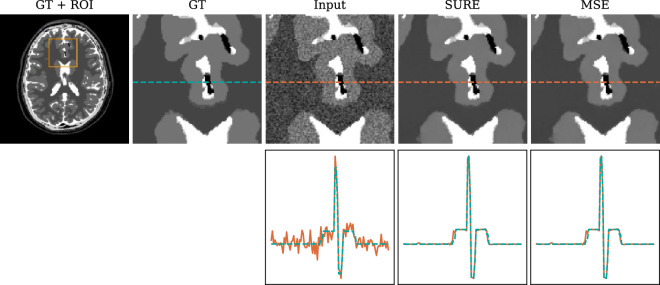
Exemplary test image that was denoised using networks trained with MSE loss with ground-truth data as well as SURE loss without ground-truth data. Both strategies are able to recover the underlying ground-truth image from the noisy input version, yielding equivalent denoising results. To evaluate edge and contrast preservation, the second row displays intensity profiles along lines drawn in the images, representing the ground truth (green) and the input/output images (orange).

The quantitative results are presented in Table 1. Although the network trained with SURE loss did not require noise-free target images, it achieved competitive performance compared to the network trained in a supervised manner with MSE loss. This finding is further supported by the qualitative evaluation presented in Fig. 4, demonstrating that both loss functions yield equivalent denoising results.

**Table 1:** Comparison of MSE and SURE loss functions for training.

Metrics	MSE	PSNR	SSIM
Input images	0.1313	25.22	0.780
SURE loss	0.0088	37.34	0.994
MSE loss	0.0088	37.36	0.994

### Training with different noise map scenarios

**Figure 5:**
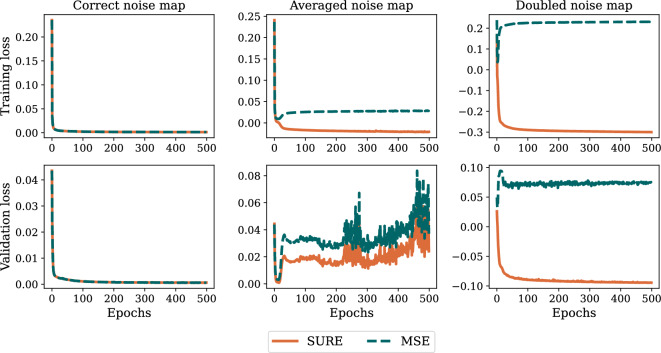
Comparison of SURE and MSE loss for different noise map scenarios. The first column displays the training and validation loss curves under the condition that the accurate noise map was used during the training process. For the second column, the noise maps were averaged and the conventional SURE approach using a scalar noise level was employed. For the third column, the noise map was doubled for SURE loss calculation without changing the noise that was actually present in the training images. The SURE loss is only capable to accurately approximate the true MSE loss if the noise map is correct (first column), otherwise SURE loss and the true MSE loss diverge (second and third column). Utilizing the averaged noise map further reveals indications of overfitting, as the validation loss increases over time.

The aim of this experiment was to investigate the influence of different noise map scenarios on the training process. With each training step, the network weights were updated backpropagating SURE loss while simultaneously tracking the true MSE between output and ground truth. Figure 5 illustrates the training loss for different noise map scenarios. When using the correct noise map, SURE and MSE are perfectly aligned. In addition, to investigate the importance of the SURE extension to spatially variant noise, we tested the original scalar noise level approach, for which we computed the average over the noise map, while the input image and actual image noise remained the same. As a result, Fig. 5 shows that the SURE and MSE loss curves diverge, with SURE even becoming negative. Note that this is possible because the divergence term can assume negative values. If we double the noise map during training without actually changing the noise present in the images, this effect is even amplified.

### Experiments on real data

**Figure 6:**
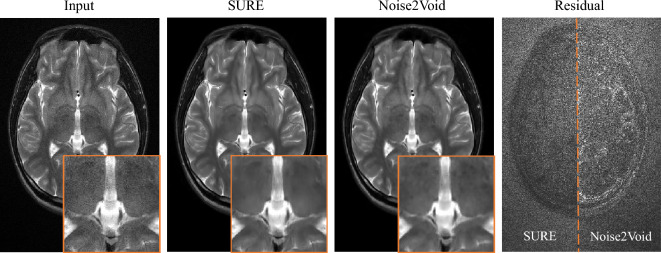
Results of denoising real MR images without ground-truth data. Our proposed SURE-based approach demonstrates superior performance compared to the reference method Noise2Void in terms of image sharpness and clarity. The residual images show the difference between the input and the denoised images, i.e., the removed noise.

The real data set was used to train a denoising network using our proposed SURE loss without any ground-truth data. The complex-valued images were fed into the network concatenated as two input channels. During training, SURE loss was then averaged over both real and imaginary images. Since the resulting denoising performance of the network could not be assessed by calculating standard quality metrics, the denoised images were visually evaluated and compared with the outcomes of a network trained using the state-of-the-art unsupervised Noise2Void principle^17^. Exemplary result magnitude images are illustrated in Fig. 6. While the SURE-based network is able to recover a sharp, noise-free image, the Noise2Void result exhibits patchy artifacts and a substantial loss in resolution. When looking at the residual images, i.e., the difference between network input and output, it becomes apparent that, in contrast to the network trained with Noise2Void, the network trained with SURE removes less signal at tissue borders and thus maintains sharp edges.

**Figure 7:**
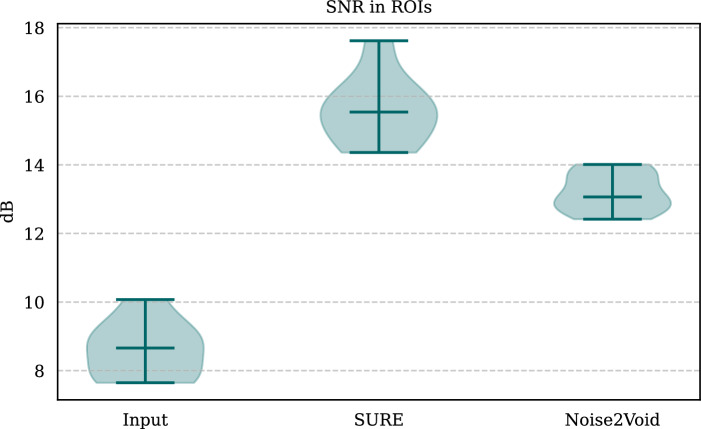
SNR assessments on small, approximately homogeneous ROIs within white and grey matter for five test slices (two ROIs per slice). The results highlight the enhanced performance of the SURE-based network compared to the network trained using the Noise2Void principle.

We further conducted SNR assessments on small, approximately homogeneous regions of interest (ROIs) within white and grey matter across five test slices, with two ROIs per slice. The outcomes are depicted in Fig. 7. Notably, our SURE-based network demonstrates superior performance compared to the network trained using the Noise2Void principle.

**Figure 8:**
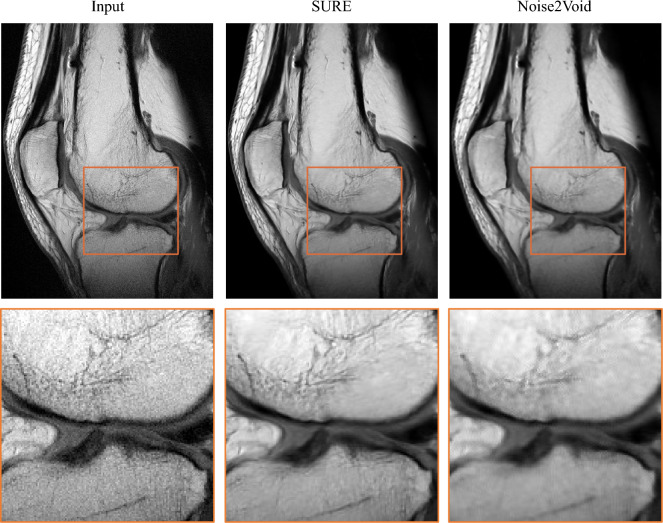
Evaluation of model robustness. The network, trained on T2-weighted 0.55 T head scans, is tested on a PD-wighted knee scan acquired at 1.5 T. The results demonstrate the adaptability of our SURE-based model in reducing image noise while preserving detailed structures and edges. In contrast, the model trained using Noise2Void predicts images with blurred fine structures and details.

### Testing model robustness

To assess the robustness of our model, we tested the network trained on T2-weighted 0.55 T head scans on a PD-weighted knee scan acquired at 1.5 T. The results are displayed in Fig. 8. Despite being trained on a different body region, field strength, and image contrast, our model demonstrates remarkable adaptability, effectively reducing image noise while preserving detailed structures and edges. In contrast, the model trained using the Noise2Void principle shows a noticeable loss of fine structures and details.

### Tuning the denoiser

**Figure 9:**
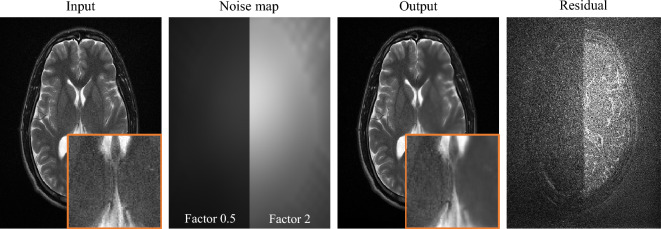
Exemplary noise map manipulation. The left side of the noise map was multiplied by a factor of 0.5, the right side by a factor of 2. The residual image shows the difference between the noisy image and the result image, i.e., the removed noise.

In this section, we demonstrate how to tune the denoiser during inference by using a model conditioned on the noise map. So far, the noise maps were only used to calculate the SURE loss during training. Similar to Zhang et al.^15^, in this experiment, the noise maps were fed into the network as an additional input channel, concatenated with the complex-valued MR images. Initially, Zhang et al.^15^ proposed to integrate noise maps as additional input channels to deal with different levels of noise in the input images. Now this idea can be taken one step further by manipulating the given noise maps during inference and thereby controlling the denoising network in a particular way. For instance, Fig. 9 demonstrates how the network output can be influenced by scaling the noise map to increase or decrease the level of denoising.

## Discussion

In this work, we demonstrated that even in the absence of noise-free ground-truth images it is possible to achieve a denoising performance that is equivalent to state-of-the-art supervised training by using SURE to estimate the true MSE. The results of the SURE and MSE-based training were competitive in both the quantitative and visual evaluation, suggesting that SURE loss approximates MSE loss reasonably well without requiring any noise-free data. Our experiments showed that the special consideration of the presence of spatially variant noise in our proposed SURE extension is important to achieve adequate denoising results, since working with an averaged noise level leads to distorted training effects.

For our experiments aiming to compare SURE and MSE as loss functions we used simulated MR images containing artificial features as ground truth to mitigate a potential influence of residual image noise in high-field ground-truth data on the quantitative and visual results. We further demonstrated the applicability of our proposed approach to real images in our experiments using data acquired at a field strength of 0.55 T, where our SURE-based loss visually outperformed state-of-the-art unsupervised denoising method Noise2Void. In our experimental evaluation using knee data, our model exhibited robust performance across varying image contrasts, field strengths, and, consequently, noise levels. This versatility underscores the model’s adaptability to diverse MR imaging scenarios.

Our SURE-based loss was computed over a set of samples during training and evaluation. Theoretically, SURE can also be used to denoise a single image without prior training^19^. However, in this case, the accuracy of SURE is not guaranteed since in theory an infinite amount of samples is needed to accurately predict the expected value of the MSE. Thus, in practice small deviations between SURE and MSE may occur also when averaging over a large data set, which can either negatively or positively affect the network performance on unseen test data.

The integration of the noise map as a third input channel is beneficial as it allows the denoising level to be adjusted as desired at inference time. This feature could be particularly interesting for customization in clinical applications to meet the distinct preferences of the radiologists. In this way, the tradeoff between denoising and image sharpness can be addressed when training with SURE loss without relying on ground-truth data. Moreover, the denoising level can be adjusted locally, e.g., for different anatomical structures.

SURE estimates the true MSE by incorporating supplementary information about the local noise level in the form of a dedicated noise map. It was shown that the use of inaccurate noise maps has a negative impact on the overall training process and thus also the denoising results. If the noise level is overestimated, the loss even drifts into the negative. Note that this is possible because the divergence term can assume negative values. For MR images, an accurate noise model can be determined considering the image reconstruction parameters^28^ and intensity inhomogeneity correction^29^, as described in “Noise estimation in MRI”.

Our proposed denoising method can be beneficial for low-field image enhancement or reducing scan times by accepting higher noise levels through, e.g., higher bandwidth imaging or fewer averages. In general, our SURE extension is only applicable to Gaussian noise. Therefore, the availability of complex-valued reconstructed images is required in the case of MRI denoising. Calculating SURE on the real and imaginary parts of the images separately mitigates the problem of dealing with complex operators. Besides MRI, the proposed method can be adopted for other modalities where a spatially variant Gaussian noise model applies.

Similar to our proposed method, diffusion models offer the advantage of denoising MRI scans without the need for paired noisy and noise-free data. However, their drawbacks include computational complexity, which can result in time-consuming processes. Despite these challenges, diffusion models have demonstrated promising results in the existing literature.

While this work focused on the denoising of already reconstructed image data, common practice in the field of MRI also applies regularization techniques to mitigate noise amplification during image reconstruction. In contrast to our proposed image-to-image denoising approach, DL-based reconstruction is a more complex task, demanding increased computational resources due to its iterative nature. However, the effectiveness of denoising an already reconstructed image might be limited in addressing noise amplification arising from the entire image acquisition process. DL-based reconstruction, on the other hand, tackles the complete image reconstruction task, potentially providing a more holistic solution to challenges associated with noisy or undersampled MRI data. Thus, the potential benefit of applying our proposed SURE loss extension for spatially variant Gaussian noise during reconstruction can be investigated in future work.

## Conclusion

In this work, SURE was adapted for MRI applications considering the spatially variant noise enhancement during parallel image reconstruction. Using the extended SURE loss and a dedicated noise model, we trained a denoising network without knowledge of the underlying noise-free ground-truth images. The proposed approach achieved competitive performance compared to state-of-the-art supervised learning using MSE loss and clearly outperformed conventional unsupervised approaches like Noise2Void. Additionally, the noise model can be used to tune the level of denoising according to the preference of the radiologist.

## Data Availability

The simulated data set that was used for the basic analysis of the proposed method is publicly available in the *BrainWeb20* MR data base^37^. The images can be generated using the *brainweb* Python package https://doi.org/10.5281/zenodo.4032893. The real data set that was used for the remaining experiments was acquired by Siemens Healthcare GmbH and is not publicly available. The data are however available from the authors upon reasonable request and with permission of Siemens Healthcare GmbH.

## Appendix: Spatially variant SURE derivation

As introduced in section “Extended SURE approach for spatially variant Gaussian noise”, noisy measurement vector ${\textbf {y}}\in {{\mathbb {R}}}^{D}$ follows a multivariate Gaussian distribution with clean image ${\textbf {x}}\in {{\mathbb {R}}}^{D}$ as mean and covariance matrix $\varvec{\Sigma }$ coming from the additive noise $\varvec{\eta }\sim \ {\mathcal {N}}(0,\varvec{\Sigma })$. We consider $\varvec{\Sigma }$ to be a diagonal matrix with entries $\Sigma _{dd} = \sigma _d^2$, where $\sigma _i$ are the components of the noise map $\varvec{\sigma } \in {{\mathbb {R}}}^{D}$. The denoising operator $f({\textbf {y}})$ can be defined as ${f({\textbf {y}})={\textbf {y}}+g({\textbf {y}})}$, with $g$ being a weakly differentiable function.

Thus, we can rewrite the expectation of the MSE as follows:

7 $$\begin{aligned} \text {E}_{{\textbf {x}}}\Big \{\ \hspace{-0.3em} \frac{1}{D} \Vert f({\textbf {y}})-{\textbf {x}}\Vert ^2 \Big \}= \text {E}_{{\textbf {x}}}\Big \{\ \hspace{-0.3em} \frac{1}{D} \Vert g({\textbf {y}})\Vert ^2 \Big \}\ + \text {E}_{{\textbf {x}}}\Big \{\ \hspace{-0.3em}\frac{1}{D} \Vert {\textbf {y}}- {\textbf {x}}\Vert ^2 \Big \}\ + \frac{2}{D}\text {E}_{{\textbf {x}}}\big \{\ g({\textbf {y}})^T({\textbf {y}}-{\textbf {x}})\big \}\ . \end{aligned}$$

By incorporating $\text {Var}({\textbf {y}}) = \text {E}({\textbf {y}}-{\textbf {x}})^2$, the second term $\text {E}_{{\textbf {x}}}\Vert {\textbf {y}}- {\textbf {x}}\Vert ^2$ can be rewritten as:

8 $$\begin{aligned} \text {E}_{{\textbf {x}}}\Big \{\ \hspace{-0.3em}\frac{1}{D} \Vert {\textbf {y}}- {\textbf {x}}\Vert ^2 \Big \}= \frac{1}{D} \sum \limits _d^D\sigma _d^2\hspace{5.0pt}. \end{aligned}$$

Including the definition of the expectation operator, the last term $\text {E}_{{\textbf {x}}} \big \{ \hspace{-0.3em} g({\textbf {y}}) ^T({\textbf {y}}-{\textbf {x}})\big \}$ can be expanded:

9 $$\begin{aligned} \text {E}_{{\textbf {x}}}\big \{\ g({\textbf {y}})^T({\textbf {y}}-{\textbf {x}})\big \}= \int _{R^D} \gamma \beta g({\textbf {y}})^T({\textbf {y}}- {\textbf {x}}) \,d^D{\textbf {y}}\end{aligned}$$

10 $$=\int _{R^D} \gamma \beta \sum \limits _d^D g_d({\textbf {y}})(y_d-x_d)\,d^D{\textbf {y}}\hspace{5.0pt}.$$

with

11 $$\begin{aligned} \gamma&= \dfrac{1}{\sqrt{(2\pi )^D \det (\varvec{\Sigma })}} \hspace{5.0pt}, \end{aligned}$$

12 $$\begin{aligned} \beta&= \exp \biggl (-\dfrac{1}{2}\Vert {\textbf {y}}- {\textbf {x}}\Vert ^T \varvec{\Sigma }^{-1}\Vert {\textbf {y}}- {\textbf {x}}\Vert \biggr ) \hspace{5.0pt}. \end{aligned}$$

Using integration by parts and the following considerations:

**Table:**

$$\begin{aligned}{}&\int uv' = \bigl [uv \bigr ]- \int u'v \\&u \hspace{0.1cm}= g_d({\textbf {y}})\\&u' = \frac{\partial u}{\partial y_d} = \frac{\partial g_d({\textbf {y}})}{\partial y_d}\\&v' = \frac{\partial v}{\partial y_d} = (y_d-x_d) \beta \\&v \hspace{0.1cm}= -\Sigma _{dd} \beta = -\sigma _d^2 \beta \\ \\&\text {Note that since}\, \varvec{\Sigma }\,\text {is a diagonal matrix, we can write: } \\&\beta = \exp \biggl (-\dfrac{1}{2}\Vert {\textbf {y}}- {\textbf {x}}\Vert ^T \varvec{\Sigma }^{-1}\Vert {\textbf {y}}- {\textbf {x}}\Vert \biggr ) = \exp \biggl (-\dfrac{1}{2}\sum \limits _j^D \frac{(y_j-x_j)^2}{\Sigma _{jj}} \biggr )\\&\Rightarrow \frac{\partial (y_j-x_j)^2}{\partial y_d}=0 \text { if } d\ne j \end{aligned}$$	

we can further rewrite Eq. (10) as:

13 $$\begin{aligned} \int _{R^D} \gamma \beta \sum \limits _d^D g_d({\textbf {y}})(y_d-x_d)\,d^D{\textbf {y}}= \sum \limits _d^D \gamma \left[ g_d({\textbf {y}})\sigma _d^2 \beta \right] _{-\infty }^\infty - \sum \limits _d^D \gamma \int \frac{\partial g_d({\textbf {y}})}{\partial y_d}(-\sigma _d^2) \beta \,d^D{\textbf {y}}\hspace{5.0pt}. \end{aligned}$$

The first term of Eq. (13) vanishes because the exponential function approaches zero when raised to the power of negative infinity, so that:

14 $$\begin{aligned} -\sum \limits _i^D\gamma \int \frac{\partial g_i({\textbf {y}})}{\partial y_i}(-\sigma _i^2) \beta \,d^D{\textbf {y}}&= \sum \limits _i^D \sigma _i^2\text {E}_{{\textbf {x}}}\frac{\partial g_i({\textbf {y}})}{\partial y_i} \end{aligned}$$

Finally, the terms can be recombined and $g({\textbf {y}})$ is replaced by $f({\textbf {y}})- {\textbf {y}}$:

15 $$\begin{aligned} \text {E}_{{\textbf {x}}}\Big \{\ \hspace{-0.3em} \frac{1}{D} \Vert f({\textbf {y}})-{\textbf {x}}\Vert ^2 \Big \}= \text {E}_{{\textbf {x}}}\Big \{\ \hspace{-0.3em} \frac{1}{D} \Bigl ( \Vert g({\textbf {y}})\Vert ^2 + \sum \limits 
_d^D\sigma _d^2 +2\sum \limits _d^D \sigma _d^2 \frac{\partial g_d({\textbf {y}})}{\partial y_d} \Bigr ) \Big \}\ \end{aligned}$$

16 $$= \text {E}_{{\textbf {x}}}\Big \{\ \hspace{-0.3em} \frac{1}{D} \Bigl ( \Vert f({\textbf {y}})- {\textbf {y}}\Vert ^2 - \sum \limits _d^D\sigma _d^2 +2\sum \limits _d^D \sigma _d^2 \frac{\partial f_d({\textbf {y}})}{\partial y_d} \Bigr ) \Big \}$$

17 $$= \text {E}_{{\textbf {x}}}\Big \{\ \hspace{-0.3em} \frac{1}{D} \Bigl ( \Vert f({\textbf {y}})-{\textbf {y}}\Vert ^2 - \sum \limits _d^D\sigma _d^2 + 2\text {div}_{{\textbf {y}}} ( \varvec{\sigma ^2} \odot f({\textbf {y}})) \Bigr ) \Big \}\ ,$$

where $\odot$ denotes element-wise multiplication.
